# Discovery of Synthetic Imine-Chalcones Targeting Mayaro Virus Replication

**DOI:** 10.3390/pathogens15050529

**Published:** 2026-05-14

**Authors:** Leonardo dos Santos Corrêa-Amorim, Natasha Cristina da Rocha, Geicy Kelly P. Barboza, Mariana F. L. P. Carlos, Aurea Echevarria, Vitor Won-Held Rabelo, Izabel Christina Nunes de Palmer Paixão

**Affiliations:** 1 Laboratório de Virologia Molecular e Biotecnologia Marinha, Programa de Pós-Graduação em Ciências e Biotecnologia, Departamento de Biologia Celular e Molecular, Instituto de Biologia, Universidade Federal Fluminense, Niterói 24210-201, RJ, Brazil; leonardoamorim@id.uff.br (L.d.S.C.-A.);; 2 Gerência de Desenvolvimento Tecnológico, Diretoria Científica, Instituto Vital Brazil, Niterói 24230-410, RJ, Brazil; 3 Instituto de Química, Departamento de Química Orgânica, Universidade Federal Rural do Rio de Janeiro, Seropédica 23897-000, RJ, Brazilechevarr@ufrrj.br (A.E.); 4 Departamento de Virologia, Instituto de Microbiologia Paulo de Góes, Centro de Ciências da Saúde, Universidade Federal do Rio de Janeiro, Rio de Janeiro 21941-902, RJ, Brazil

**Keywords:** arboviruses, Mayaro virus, chalcone, flavonoid, antiviral

## Abstract

Mayaro virus (MAYV) is an arthritogenic alphavirus transmitted by mosquitoes and is the causative agent of Mayaro fever. This disease is associated with symptoms such as arthralgia and myalgia, which may persist for months or even years. Currently, no vaccine or specific antiviral therapy is available. This study aimed to assess the antiviral activity of synthetic imine-chalcone derivatives (**1a**–**1d**) against MAYV replication in Vero cells and predict their pharmacokinetic and toxicological properties. All compounds presented low cytotoxicity, with CC_50_ values ranging from 249.92 µM to >1000 µM. Additionally, the derivatives showed good antiviral activity, with compound **1a** being the most potent (EC_50_ = 12.15 μM; SI = 31.47), and **1b** being the most selective (EC_50_ = 16.92 μM; SI > 59.10). Mechanistic assays revealed that compounds **1a** and **1b** primarily inhibit early events in the MAYV life cycle, such as viral adsorption (**1a**: 51.53%; **1b**: 59.35%) and entry (**1a**: 71.26%; **1b**: 54.21%). Compound **1b** also impaired virus egress, while none of the compounds exhibited strong virucidal activity. Finally, in silico ADMET predictions suggested favorable pharmacokinetic and toxicological parameters for compounds **1a** and **1b**. Overall, our work demonstrated for the first time the activity and safety of imine-chalcones against MAYV.

## 1. Introduction

Human behavior and technological advances have led to significant changes on the planet, including climate change, deforestation, and globalization, which may increase human contact with emerging and re-emerging pathogens [1,2]. In this scenario, arboviruses are viruses transmitted by arthropods and pose a serious threat to human health. While over one hundred arboviruses have been identified as human pathogens, the most relevant ones belong to only a few families, like Flaviviridae and Togaviridae [3]. Among them, Mayaro virus (MAYV) is an enveloped virus of increasing concern that belongs to the Togaviridae family and the *Alphavirus* genus. This virus has a positive-sense single-stranded RNA genome of approximately 11.5 kb that encodes five structural proteins and peptides (capsid, envelope glycoproteins E1, E2, E3, and 6k/transframe) and four non-structural proteins (nsP1-4) [4].

MAYV is mainly transmitted by *Haemagogus* spp. mosquitoes and was first isolated in 1954 in Trinidad and Tobago [5]. Since then, more than 10 Latin American countries have reported cases of autochthonous transmission, and outbreaks have been confirmed in at least five countries, with Brazil holding the highest number of cases. Although this virus is prevalent in Latin America, imported cases have already been reported in the United States of America (USA) and European countries [6]. Additionally, MAYV likely circulates among non-human primates and other vertebrate hosts, and since anthropophilic mosquitoes are potential vectors (e.g., *Aedes* and *Anopheles* spp.), this virus has the potential to emerge as a global pathogen [5,7].

Clinically, MAYV is responsible for a mild and self-limited febrile disease characterized by fever, myalgia, arthralgia, headache, and rash. However, more severe manifestations, such as neurological disorders, hemorrhage, and myocarditis, may occur less frequently [8]. Moreover, in regions such as Brazil, where other arboviruses circulate, such as Chikungunya virus (CHIKV), Zika virus (ZIKV), and Dengue virus (DENV), coinfection with MAYV and other viruses, particularly CHIKV, has been confirmed and associated with even more severe symptoms [9,10]. Worryingly, as with other arthritogenic alphaviruses, arthralgia can persist for years in nearly 50% of patients, which drastically compromises their quality of life and leads to critical economic losses [11].

The overlapping symptoms of cocirculating arboviruses (e.g., CHIKV, DENV, and ZIKV) combined with limited diagnostic testing capacity hinder disease control efforts [3]. Despite ongoing efforts, there are currently no vaccines or antiviral therapies available for the treatment or prevention of this viral infection [12]. Therefore, the search for new therapies is urgently needed.

In this context, chalcones stand out as a privileged scaffold of great importance in pharmaceutical research. Structurally, chalcones, also known as 1,3-diaryl-2-propen-1-ones, bear an α, β-unsaturated carbonyl group that links two phenyl rings. These compounds occur naturally and represent one of the major classes of secondary metabolites in plants [13,14]. In addition, low-cost synthetic methods for chalcones with high yields have been described, which, in turn, allow the generation of chemically diverse derivative libraries and provide a highly versatile scaffold for the development of new bioactive compounds [15].

Chalcones are endowed with a wide range of biological properties, such as geroprotective and neuroprotective effects [16,17], as well as antidiabetic, anti-inflammatory, antioxidant, antitumoral, antiparasitic [18], antimicrobial [19], and antiviral [20] activities. Our research group has also designed several chalcone derivatives with interesting pharmacological activities, including anticancer [21], antileishmanial [22], and antifungal [23]. In addition, we have designed and synthesized three imine-chalcones with demonstrated antifouling activity [24]. However, the pharmacological potential of this scaffold, particularly its antiviral effects, remains unknown. Thus, in this study, novel imine-chalcone derivatives were designed, synthesized, and their antiviral profile was evaluated in vitro against MAYV. We further investigated their pharmacokinetic and toxicological properties using in silico methods to assess their potential as future drug candidates.

## 2. Materials and Methods

### 2.1. Synthesis

#### 2.1.1. General

Reagents and solvents were purchased from commercial sources and were used without previous purification. NMR spectra were acquired on a Bruker Avance 500 Ultra Shield (Bruker BioSpin GmbH & Co. KG, Berlin, Germany) spectrometer operating at 500 MHz (1H) and 125 MHz (13C), using DMSO-d6 or CDCl3 and TMS as internal references. FT-IR spectra were obtained on a Bruker VERTEX 70 (Bruker Optics GmbH & Co. KG, Karlsruhe, Germany) spectrometer using the ATR method. Elemental analyses were conducted using a PerkinElmer 2400 CHN (PerkinElmer, Inc., Shelton, CT, USA) instrument in the Laboratory of Environmental Science at the State University of Northern Rio de Janeiro (UENF). Thin-layer chromatography (TLC) analyses were conducted on silica gel 60 F254 (Merck KGaA, Darmstadt, Germany) plates.

#### 2.1.2. Synthetic Procedure

The imine-chalcones were synthesized by adding 0.5 mol of phenethylamine to a round-bottom flask containing 0.5 mol of chalcone, previously prepared from acetophenone and 4-halogenated benzaldehyde in an alkaline medium, in ethanol as the solvent, in a scientific microwave oven at 78 °C and 100 W for 40 min. After the mixture was evaporated, 50 mL of CH_2_Cl_2_ was added_;_ the mixture was washed with water (3 × 20 mL), dried over anhydrous sodium sulfate, filtered, and concentrated to obtain the imine-chalcones as oils, which were subsequently purified by filtration into filtrates and on silica gel.

*[(1Z,2E)-1,3-diphenylprop-2-en-1-ylidene]-2-phenetylamine* (**1a**). Yield: 82%; light yellow oil; refractive index: 1.600; FT-IR (ATR, ν, cm^−1^): 3025, 2848, 1645, 1600, 1450; ^1^H NMR (500 MHz, CDCl_3_) δ 8.20 (m, 1H, Ar-H), 7.56 (m, 1H, CH), 7.23 (m, 1H, Ar-H), 6.43 (d, 1H, *J* = 5 Hz, CH_2_), 3.56 (t, 2H, *J* = 8 Hz, CH_2_), 3.05 (t, 2H, *J* = 8, CH_2_); ^13^C NMR (125 MHz, CDCl_3_) δ 166.7, 142.9, 141.7, 137.9, 135.9, 130.1, 125.8, 58.8, 36.9. Anal. calc. for C_23_H_21_N: C, 88.71; H, 6.80; N, 4.50. Found: C, 88.74; H, 6.76; N, 4.51.
*[(1Z,2E)-3-(4-chlorophenyl)-1-phenylprop-2-en-1-ylidene]-2-phenethylamine* (**1b**). Yield: 93%; dark brown oil; refractive index: 1.598; FT-IR (ATR, ν, cm^−1^): 3027, 2863, 1643, 696; ^1^H NMR (500 MHz, CDCl_3_) δ 8.03 (m, 1H, Ar-H), 7.41 (m, 1H, CH), 7.15 (m, 1H, Ar-H), 6.70 (d, 1H, *J* = 5 Hz, CH_2_), 3.64 (t, 2H, *J* = 8 Hz, CH_2_), 3.14 (t, 2H, *J* = 8 Hz, CH_2_); ^13^C NMR (125 MHz, CDCl_3_) δ 160.0, 139.8, 138.2, 137.4, 136.6, 134.6, 126.2, 62.9, 37.2. Anal. calc. for C_23_H_20_ClN: C, 79.87; H, 5.83; N, 4.05. Found: C, 79.91; H, 5.81; N, 3.98.
*[(1Z,2E)-3-(4-bromophenyl)-1-phenylprop-2-en-1-ylidene]-2-phenethylamine* (**1c**). Yield: 89%; yellow oil; refractive index: 1.592; FT-IR (ATR, ν, cm^−1^): 3043, 2875, 1612, 695; ^1^H NMR (500 MHz, CDCl_3_) δ 7.45 (m, 1H, Ar-H), 7.69 (m, 1H, Ar-H), 7.39 (m, 1H, CH), 6.71 (d, 1H, *J* = 5 Hz, CH_2_), 3.65 (t, 2H, *J* = 8 Hz, CH_2_), 3.12 (t, 2H, *J* = 8 Hz, CH_2_); ^13^C NMR (125 MHz, CDCl_3_) δ 160.0, 139.5, 135.2, 134.8, 133.0, 125.9, 124.7, 62.8, 37.1. Anal. calc. for C_23_H_20_BrN: C, 70.78; H, 5.16; N, 3.59. Found: C, 70.73; H, 5.09; N, 3.54.
*[(1Z,2E)-3-(4-fluorophenyl)-1-phenylprop-2-en-1-ylidene]-2-phenethylamine* (**1d**). Yield: 72%; brown oil; refractive index: 1.569; FT-IR (ATR, ν, cm^−1^): 3010, 2877, 1616, 1187; ^1^H NMR (500 MHz, CDCl_3_) δ 8 (m, 1H, Ar-H), 7.52 (m, 1H, CH), 7.27 (m, 1H, Ar-H), 6.68 (m, 1H, CH_2_), 3.82 (t, 2H, *J* = 8 Hz, CH_2_), 3.02 (t, 2H, *J* = 8 Hz, CH_2_); ^13^C NMR (125 MHz, CDCl_3_) δ 165.1, 163.6, 140.1, 139.0, 137.0, 133.7, 116.0, 63.3, 37.7. Anal. calc. for C_23_H_20_FN: C, 83.86; H, 6.12; N, 4.25. Found: C, 83.82; H, 6.01; N, 4.19.

### 2.2. In Vitro Assays

#### 2.2.1. Cell Culture and Virus

Vero cells (ATCC CCL-81) were provided by the Laboratory of Viral Vaccines, Biodrugs, and Cell Culture, Department of Immunology, National Institute for Quality Control in Health, Fiocruz, Rio de Janeiro, RJ, which certified their authenticity. Cells were maintained in Dulbecco’s modified Eagle Medium (DMEM) supplemented with 5% fetal bovine serum (FBS), 100 U/mL penicillin, 100 µg/mL streptomycin, and 2.5 µg/mL amphotericin B and cultured at 37 °C under a 5% CO_2_ humid atmosphere unless otherwise stated. The MAYV TR 4675 strain (ATCC VR-66; GenBank accession code MK070492) was kindly provided by Dr. Davis Fernandes Ferreira from the Department of Virology, Federal University of Rio de Janeiro, Brazil. Virus stocks were prepared in Vero cells using a multiplicity of infection (MOI) of 0.1. At 72 h post-infection (hpi), cells were lysed by three cycles of freezing and thawing, centrifuged at 1500 rpm for 3 min, and supernatants were collected and stored at −80 °C until use.

#### 2.2.2. Cytotoxicity Evaluation

The cytotoxicity of the compounds was analyzed by measuring the cell metabolic activity with the MTT method. Vero cells were seeded in 96-well plates at a density of 2 × 10^4^ cells/well 24 h before the assay. Then, cells were treated with different concentrations of the compounds (62.5, 125, 250, 500, and 1000 µM) for 72 h at 37 °C in a 5% CO_2_ atmosphere. After treatment, the medium containing the compounds was discarded, and 50 µL of MTT solution (2 mg/mL in DMEM without FBS) was added to each well. Cells were further incubated for 4 h protected from the light, followed by the removal of supernatant and solubilization of formazan crystals with DMSO. Finally, plates were gently rocked for 20 min, and the absorbance of each well was recorded at a wavelength of 540 nm using a microplate reader (Loccus LMR-96, Cotia, SP, Brazil). The absorbance of untreated cells was considered as 100% cell viability. The assays were conducted three times independently in triplicate. The concentration needed to decrease 50% of cell viability (CC_50_) was estimated by linear regression from dose–response curves obtained with the GraphPad Prism 8.0 program (GraphPad, San Diego, CA, USA).

#### 2.2.3. Determination of the Antiviral Activity Against MAYV

The antiviral activity of the compounds was evaluated using a virus yield reduction assay [25]. Vero cells (2 × 10^5^ cells/well) cultured in 24-well plates were infected with MAYV at an MOI of 0.1 for 1 h at 37 °C in a 5% CO_2_ atmosphere. After adsorption, the virus inoculum was removed, fresh media containing compounds were added, and cells were incubated for 24 h under the same conditions. Then, cells were lysed by freezing and thawing, and the total content was harvested for virus quantification.

Virus titration was performed using standard plaque assays with Vero cells. Confluent monolayers of Vero cells were infected with serial dilutions (1:10) of the collected samples. The adsorption was allowed to occur for 1 h at 37 °C in a 5% CO_2_ atmosphere. After this, the virus inoculum was discarded, cells were covered with DMEM supplemented with 5% FBS and 1.5% methylcellulose and incubated for 72 h under the same conditions. At 72 hpi, the medium was removed, and cells were fixed and stained with 10% formaldehyde and 0.2% crystal violet. Virus titers were quantified as plaque-forming units (PFU)/mL.

First, compounds were screened for their antiviral activity at 50 µM. To determine the antiviral potency, different concentrations (6.25, 12.5, 25, and 50 µM) of the chalcone derivatives were assessed. We included suramin as a positive control because its in vitro anti-MAYV activity has been described earlier [26]. Based on this study, suramin was initially evaluated at 200 µM. Then, the antiviral potency of suramin was assessed using different concentrations, such as 12.5, 25, 100, and 200 µM. Cells infected and treated with DMSO were also included as a solvent control, and no significant inhibitory effects were observed. Inhibition rates were calculated relative to the infected and untreated group (virus control; VC). The experiments were performed in triplicate in three independent runs. The concentration required to decrease 50% of virus yield (EC_50_) was calculated by non-linear regression from dose–response curves using the GraphPad Prism 8.0 program. The selectivity index (SI) of the compounds was calculated as the ratio between CC_50_ and EC_50_ values.

#### 2.2.4. Time-of-Drug-Addition Assay

The time-of-drug-addition assay was conducted with the most active derivatives (**1a** and **1b**) according to Langendries and coworkers [26] with a few modifications. Vero cells were plated in 24-well plates at a density of 2 × 10^5^ cells/well for 24 h at 37 °C in a 5% CO_2_ atmosphere before the experiment. Cells were infected with MAYV (MOI = 1.0) for 1 h, and then, the virus inoculum was removed. Cells were treated (50 µM) at different times during infection, as follows: (a) cells were treated for 2 h before infection (−2 hpi); (b) during infection (0 hpi); and (c) at different times post-infection (2, 4, 6, and 8 hpi). In the pre-treatment group (−2 hpi), compounds were removed before infection. In the other conditions (b and c), compounds were maintained in culture medium until the end of the assay. At 10 hpi, cells were lysed by three cycles of freezing-thawing, the total content was collected, and virus titers were quantified by plaque assays. Suramin (50 µM) and DMSO (0.2%) were included as controls. Inhibition rates were determined in relation to the infected and untreated group (VC). The experiment was run thrice independently in triplicate.

#### 2.2.5. Virus Adsorption Inhibition Assay

The adsorption inhibition assay was carried out as described elsewhere [27] with a few modifications. Vero cells cultured in 24-well plates (2 × 10^5^ cells/well) were chilled at 4 °C for 1 h. Then, cells were infected with MAYV (MOI 1.0) and simultaneously treated with the most active compounds **1a** and **1b** (50 µM) for 1 h at 4 °C. DMSO (0.2%) and suramin (50 µM) controls were included in this assay. After adsorption, the virus inoculum was removed, and cells were washed twice with ice-cold phosphate saline buffer (PBS; pH 7) to remove the unbound viruses and remaining compounds. Finally, fresh media without compounds were added, and cells were incubated for 24 h at 37 °C in a 5% CO_2_ atmosphere. At 24 hpi, cells were lysed by freezing and thawing, the total content was harvested, and virus titers were quantified by plaque assays. Adsorption inhibition rates were calculated relative to the infected and untreated cells (VC group). This assay was conducted in triplicate in three independent experiments.

#### 2.2.6. Virus Entry Inhibition Study

The virus entry inhibition assay was conducted as described earlier by our group [28]. Briefly, Vero cells (2 × 10^5^ cells/well) maintained in 24-well plates were chilled for 1 h at 4 °C, followed by MAYV infection (MOI 1) for 1 h under the same conditions. After adsorption, viral inoculum was removed, cells were washed twice with ice-cold PBS (pH 7), and fresh media containing the compounds **1a**, **1b** (50 µM), SUR (50 µM), or DMSO (0.2%) were added. Cells were further incubated for 1 h at 37 °C under a 5% CO_2_ humidified atmosphere to allow viral entry. Subsequently, compounds were removed, and cells were washed with acidic PBS (pH 3), followed by neutralization with PBS (pH 7) washing. Finally, fresh media without compounds were added to the cells, which were further incubated at 37 °C and 5% CO_2_. At 24 hpi, cells were lysed by three cycles of freezing and thawing, and the total content was harvested for plaque assays. Entry inhibition rates were calculated relative to the infected and untreated control. This experiment was run thrice in triplicate.

#### 2.2.7. Virus Release Inhibition Assay

To evaluate the effects of the most active compounds **1a** and **1b** on the release of new virions, we performed a similar assay as reported by Fox and coworkers [27], with a few modifications. Vero cells previously cultivated in 24-well plates at a density of 2 × 10^5^ cells/well were infected with MAYV (MOI 1) for 2 h at 37 °C under a 5% CO_2_ atmosphere. Posteriorly, viral inoculum was removed, cells were thoroughly washed with PBS (pH 7), and fresh media containing the compounds (50 µM) were added to the cells. As controls, infected and untreated cells or cells treated with SUR (50 µM) or DMSO (0.2%) were included. Cells were further incubated for 6 h under the same conditions, allowing the first round of virus release for known alphaviruses. At 6 hpi, supernatants were collected to quantify the released infectious virus particles. The remaining cells were washed twice with PBS (pH 7), and fresh media were added to the cells, which were further lysed by freeze–thaw cycles to harvest virus particles associated with cells. Virus titers were determined by plaque assays, and inhibition rates were calculated in comparison to the infected and untreated groups. The intracellular/extracellular ratio (I/E ratio) was calculated as the ratio of virus titers in intracellular (cell-associated viruses) and extracellular (released viruses) contents. The experiments were carried out in triplicate three times independently.

#### 2.2.8. Virucidal Activity Evaluation

The virucidal activity of the most active compounds (**1a** and **1b**) was evaluated as reported previously [29] with a few modifications. MAYV suspensions (10^5^ PFU) prepared in DMEM without FBS were treated with compounds (50 µM) for 1 h at 37 °C. SUR (50 µM) and DMSO (0.2%) were included as controls. Then, suspensions were diluted (1:500) to prevent any effects on virus replication during plaque assays. The virus titer of the diluted suspensions was determined by plaque assays with Vero cells. This experiment was carried out three times in triplicate.

#### 2.2.9. Statistical Analysis

The in vitro data are expressed as mean ± standard deviation from different replicates. Statistical analysis was carried out with the GraphPad Prism 8.0 program and the one-way analysis of variance (ANOVA) method, followed by the Tukey post-test were employed for comparison across the experimental groups. Statistical significance was considered when the *p*-value ≤ 0.05.

### 2.3. In Silico Prediction

Pharmacokinetic, toxicological, and drug-like properties of the most active derivatives (**1a** and **1b**) were evaluated using computational tools. Suramin was also included as a reference drug for comparison purposes.

The SwissADME webserver [30] was used to predict pharmacokinetic parameters, such as human intestinal absorption (HIA), and blood–brain barrier (BBB) permeability, while the admetSAR 2 webserver [31] was employed to predict whether these compounds could inhibit human CYP450 isoforms (e.g., CYP2C9, CYP2C19, CYP2D6, CYP3A4). Toxicological risks for human health, such as mutagenicity, carcinogenicity, and immunotoxicity, were predicted with the ProTox-II webserver [32], whereas hepatotoxicity and cardiotoxicity (based on hERG I inhibition) were analyzed using the pkCSM webserver [33]. The nephrotoxic potential of these compounds was also predicted using the admetSAR 2 server.

Furthermore, several stereoelectronic features of these compounds were calculated using the SwissADME webserver, such as molecular weight (MW), n-octanol-water partition coefficient (cLogP), number of hydrogen bond donor and acceptor groups (HBD and HBA, respectively), number of rotatable bonds (RotB), and topological polar surface area (tPSA). Based on these features, their drug-like profile was assessed according to pharmaceutical industry rules, like Lipinski’s “rule of five” [34], Veber rule [35], and GlaxoSmithKline (GSK) 4/400 [36].

## 3. Results

### 3.1. Synthesis of Imine-Chalcone Derivatives

**Figure 1:**
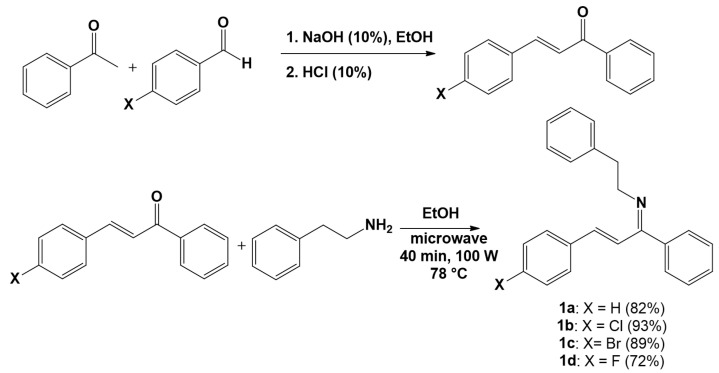
Reactions involved in the synthesis of the imine-chalcone derivatives evaluated.

The synthesis of imine-chalcones was carried out from the chalcones previously synthesized using the conventional Claisen-Schmidt condensation methodology [24], then treated with reaction with phenethylamine, through an eco-friendly methodology, using microwave irradiation, in the presence of ethanol as solvent. The reactions involved in the synthetic procedure are shown in Figure 1. The products were obtained as oils, in reaction times of 40 min with good yields (72–93%).

### 3.2. Cytotoxicity and Antiviral Activity of Synthetic Imine-Chalcone Derivatives

To evaluate the cytotoxicity of the synthesized compounds on Vero cells, the imine-chalcone derivatives (**1a**–**1d**) and the drug suramin were tested in several concentrations. The compounds **1b** and **1d** presented the lower cytotoxicity (CC_50_ > 1000 µM) as seen for the control suramin. The other compounds presented higher cytotoxicity with CC_50_ values of 382.37 µM and 249.92 µM for compounds **1a** and **1c**, respectively (Table 1).

**Figure A1:**
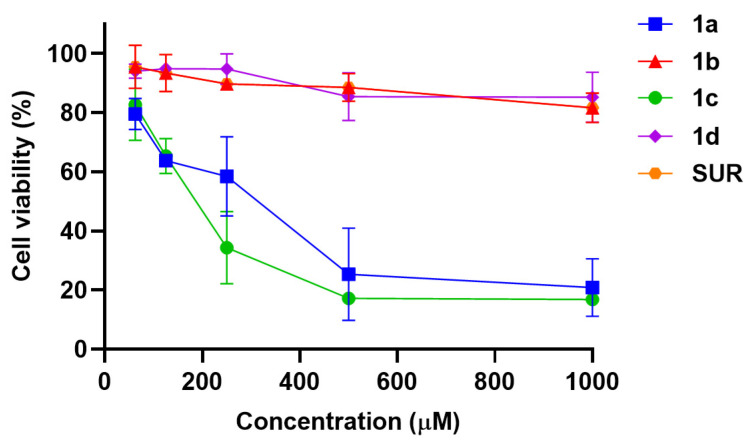
Cytotoxicity evaluation of the imino-chalcone derivatives (**1a**–**d**) and the reference drug suramin (SUR). Vero cells (2 × 10^4^ cells/well) were treated for 72 h with increasing concentrations of chalcone derivatives or SUR (62.5, 125, 250, 500, and 1000 μM). MTT assay was used to determine cell viability after treatment. Data are represented as mean ± standard deviation (n = 3).

**Figure A2:**
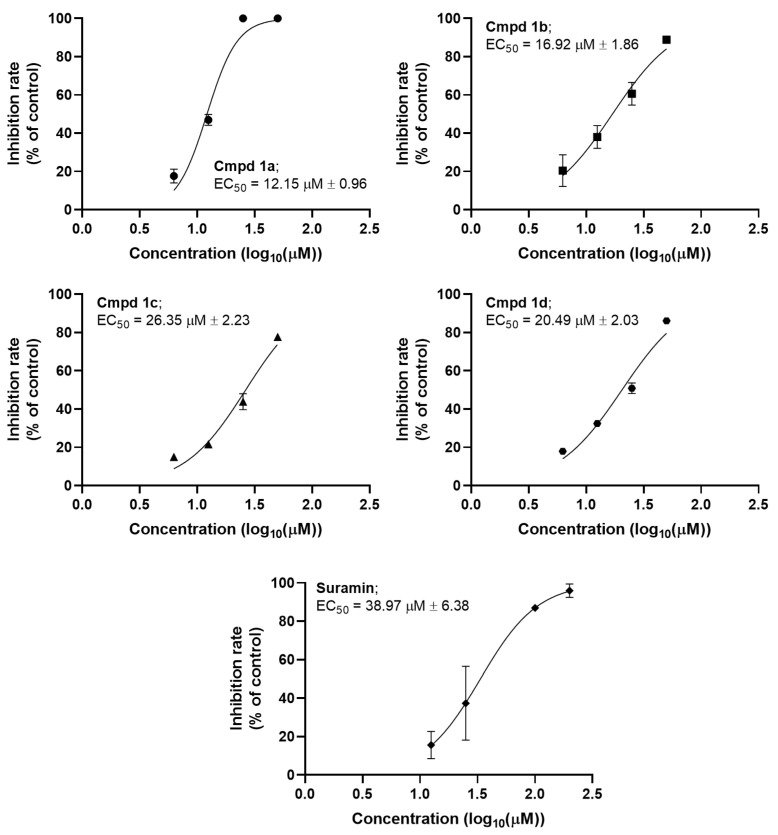
Dose–response inhibition curves of the imino-chalcone derivatives (**1a**–**d**) and the reference compound suramin against MAYV. Vero cells (2 × 10^5^ cells/well) were infected with MAYV (MOI 0.1) and treated for 24 h with increasing compound concentrations (Chalcones at 6.25, 12.5, 25, and 50 μM; Suramin at 12.5, 25, 100, and 200 μM). Virus yield was quantified by plaque assay, and inhibition rates were calculated relative to the virus control. Curves were generated by nonlinear regression, and EC_50_ values represent the concentration required to reduce virus yield by 50%. All experiments were performed in triplicate across three independent biological replicates. Error bars represent standard deviations.

After the cytotoxic profile was assessed, the screening of the imine-chalcones against MAYV was performed at a concentration of 50 μM, while the control drug suramin was used at 200 μM. All compounds exhibited strong inhibition of MAYV replication (Table 1). Compound **1a** presented the strongest inhibition rate (100%), followed by derivatives **1b** (88.91%), **1d** (86.12%), and **1c** (77.71%), whereas suramin showed an inhibition rate of 95.96%. It is important to note that the compounds showed no significant toxicity at the lowest concentration tested in cytotoxicity assays (62.5 µM) (Figure A1), indicating that the observed antiviral activity is not due to cytotoxic effects. Consequently, we determined the EC_50_ values for the imine-chalcones from dose–response curves (Figure A2). Compound **1a** presented the highest potency with an EC_50_ value of 12.15 µM, followed by **1b** (16.92 µM), **1d** (20.49 µM), and **1c** (26.35 µM) (Table 1). Besides a strong activity, the compound **1b** presented a remarkable selectivity with an SI value higher than 59.10, whereas the SI value obtained for suramin was higher than 25.66.

### 3.3. Time-of-Drug-Addition Analysis

**Figure 2:**
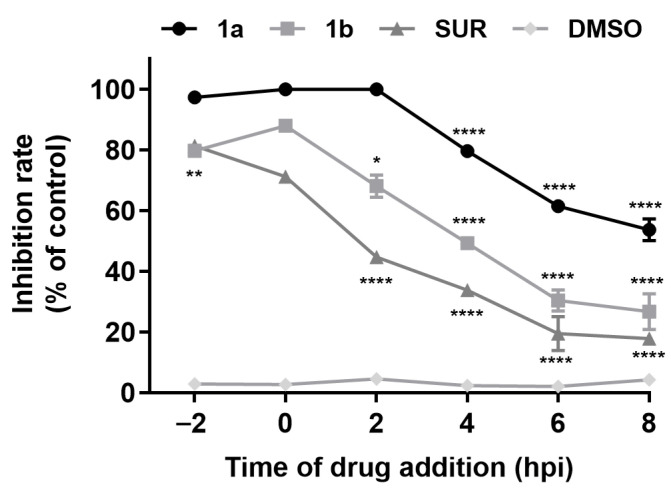
Time-of-drug-addition assay of chalcone derivatives **1a** and **1b** against MAYV replication. Vero cells (2 × 10^5^ cells/well) were infected with MAYV (MOI 1.0), and compounds were added (50 μM) at distinct time points relative to infection: 2 h before infection (−2 hpi), during virus adsorption (0 hpi), or at 2, 4, 6, and 8 h post-infection (hpi). For the pre-treatment condition (−2 hpi), compounds were removed before viral adsorption; in all other conditions, compounds remained present until the end of the assay. At 10 hpi, cells were lysed, and virus yields were quantified by plaque assay. Suramin (50 μM) and DMSO (0.2%) were used as controls. Inhibition rates were calculated relative to the virus control. Data represent mean ± SD from three independent experiments performed in triplicate. Statistical analysis was performed using 0 hpi as the reference time point, and the significance threshold was defined as *p*-value < 0.05 (*), *p* < 0.01 (**) and *p* < 0.0001 (****).

After the preliminary evaluation of the anti-MAYV activity of the imine-chalcones, compounds **1a** and **1b** were selected for further studies due to their antiviral activity and selectivity. To characterize their mechanism of action, a time-of-drug-addition assay was first performed (Figure 2). Compound **1a** exhibited a pronounced inhibitory effect when added up to 2 hpi, achieving ~100% inhibition during the pre-treatment (−2 hpi), entry (0 hpi), and early post-entry (2 hpi) phases. At later time points (4–8 hpi), its activity gradually declined to inhibition rates of 79.58% (4 hpi), 61.46% (6 hpi), and 53.69% (8 hpi), indicating that **1a** predominantly targets early steps of the MAYV replication cycle. As well as **1a**, compound **1b** significantly inhibited virus replication when added as a pre-treatment and post-infection up to 2 hpi with inhibition rates of 79.70% (−2 hpi), 87.91% (0 hpi), and 68.08% (2 hpi). Although both compounds exhibited a similar trend of gradual reduction in activity, compound **1b** showed a more pronounced reduction in inhibitory activity when added after 4 hpi, with inhibition rates dropping below 50% (Figure 2).

### 3.4. Inhibition of Viral Adsorption by ***1a*** and ***1b*** in Vero Cells

**Figure 3:**
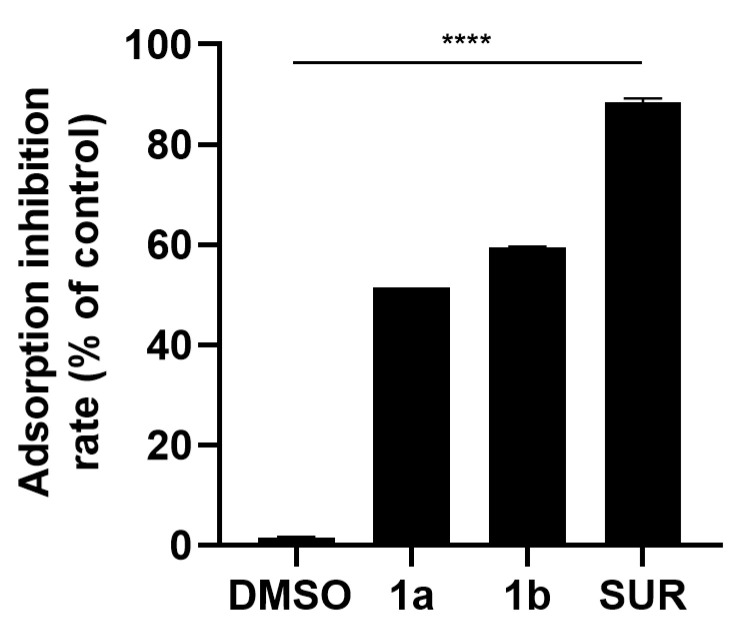
Adsorption inhibition activity of chalcone derivatives **1a** and **1b** against MAYV. Vero cells (2 × 10^5^ cells/well) were pre-chilled at 4 °C and simultaneously exposed to MAYV (MOI 1.0) and each compound (50 μM) for 1 h at 4 °C to block viral internalization. After the adsorption period, cells were washed with ice-cold PBS to remove unbound virions and residual compounds, overlaid with fresh compound-free medium, and incubated for 24 h at 37 °C. Virus yields were quantified by plaque assay, and adsorption inhibition rates were calculated relative to the virus control. Suramin (50 μM) and DMSO (0.2%) served as controls. Data represent mean ± SD from three independent experiments performed in triplicate. Statistical significance was determined relative to the DMSO control, and the significance threshold was defined as *p*-value < 0.0001 (****).

Since compounds **1a** and **1b** exhibited high activity during the early stages of MAYV infection, we evaluated their effects on viral adsorption specifically. Both compounds significantly reduced MAYV adsorption on Vero cells, with inhibition rates of 51.53% for compound **1a** and 59.35% for compound **1b**. Suramin showed an inhibition of 88.44% (Figure 3).

### 3.5. Inhibition of MAYV Entry into Vero Cells

**Figure 4:**
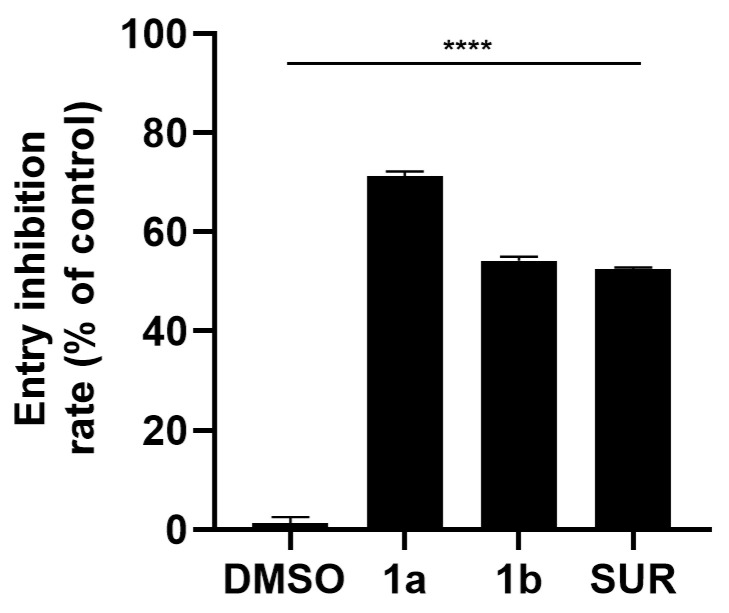
Entry inhibition activity of chalcone derivatives **1a** and **1b** against MAYV. Vero cells (2 × 10^5^ cells/well) were pre-chilled at 4 °C and infected with MAYV (MOI 1.0) for 1 h at 4 °C. After viral adsorption, cells were washed with ice-cold PBS and exposed to each compound (50 μM) for 1 h at 37 °C to allow synchronized viral entry. Compounds were then removed, cells were washed with acidic PBS (pH 3) to inactivate non-internalized virions, neutralized with PBS (pH 7), and overlaid with fresh compound-free medium. At 24 hpi, cells were lysed, and viral titers were quantified by plaque assay. Entry inhibition rates were calculated relative to the virus control. Suramin (SUR, 50 μM) and DMSO (0.2%) were included as controls. Data represent mean ± SD from three independent experiments performed in triplicate. Statistical analysis was performed using the DMSO control as the reference, and the significance threshold was defined as *p*-value < 0.0001 (****).

After the adsorption step, the inhibitory activity of both imine-chalcones on the MAYV entry was evaluated. Compounds **1a** and **1b** significantly reduced viral entry, with inhibition rates of 71.26% and 54.21%, respectively. Suramin displayed a similar inhibitory profile to compound **1b**. In general, compound **1a** displayed the strongest activity, exceeding both **1b** and the control drug, highlighting the potency of these imine-chalcones during the entry step of infection (Figure 4).

### 3.6. Effects of Chalcone Derivatives on Viral Release Dynamics

**Figure 5:**
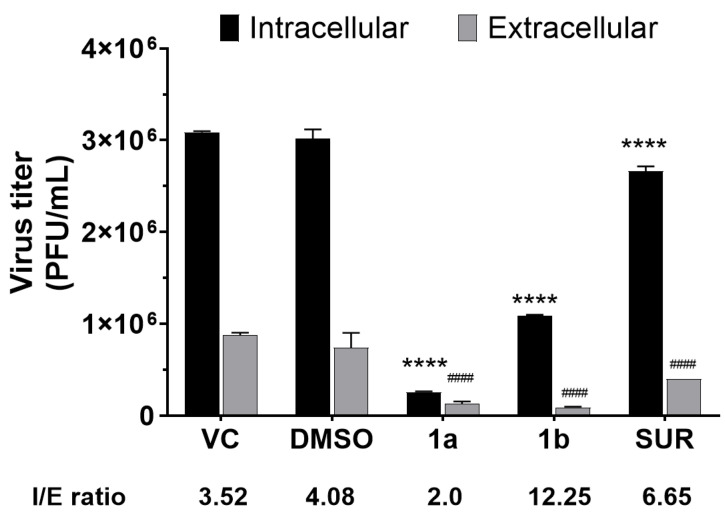
Effect of chalcone derivatives **1a** and **1b** on MAYV release. Vero cells (2 × 10^5^ cells/well) were infected with MAYV (MOI 1.0) for 2 h at 37 °C. After removal of the inoculum and PBS washing, cells were treated with compounds **1a** or **1b** (50 µM). Suramin (50 µM) and DMSO (0.2%) were included as controls. Viral titers were determined by plaque assays, and the intracellular/extracellular (I/E) ratio was calculated as the ratio between cell-associated and released infectious viral particles. Data represent mean ± SD from three independent experiments performed in triplicate. Statistical significance was determined relative to the virus control; the significance threshold was defined as *p*-value < 0.0001 (****) for comparison of virus titers in the intracellular content and *p*-value < 0.0001 (####) for comparison of virus titers found in the extracellular content.

Since imine-chalcones were able to inhibit late stages of MAYV replication, we assessed their effects on viral egress. Compound **1a** reduced both intracellular and extracellular titers with an I/E ratio = 2.0, which is lower than that of the virus control (I/E ratio = 3.52) or DMSO vehicle control (I/E ratio = 4.08) (Figure 5). In contrast, compound **1b** showed a higher I/E ratio (12.25), indicating a strong inhibition of viral egress. Suramin (I/E ratio = 6.65) exhibited a similar inhibitory effect on viral egress, but weaker than compound **1b** (Figure 5).

### 3.7. Virucidal Effect of the Most Active Derivatives on Infectious MAYV Particles

**Figure 6:**
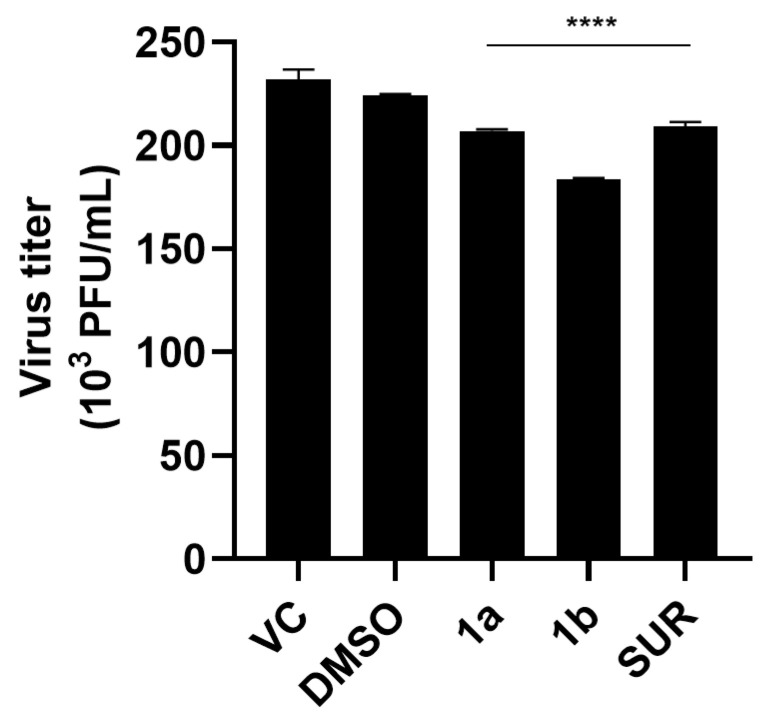
Virucidal activity of chalcone derivatives **1a** and **1b** against MAYV. MAYV suspensions (10^5^ PFU) were incubated with each compound (50 μM) for 1 h at 37 °C in serum-free DMEM. Following incubation, mixtures were diluted 1:500 to prevent compound-mediated effects during titration, and residual viral infectivity was quantified by plaque assay in Vero cells. Suramin (50 μM) and DMSO (0.2%) were used as controls. Data represent mean ± SD from three independent experiments performed in triplicate. Statistical analysis was performed using the virus control as the reference, and the significance threshold was defined as *p*-value < 0.0001 (****).

Besides evaluating the effects of the most active chalcone derivatives on different steps of virus replication, we also investigated their direct effects on viral particles (virucidal activity). Although a statistically significant reduction in viral titer was observed compared to the untreated control, virucidal activity was low. Compounds **1a** and **1b** decreased virus infectivity by 10.64% and 20.60%, respectively. Suramin, used as a reference compound, showed an inhibition value of 9.70%, while the DMSO control remained low (3.09%) (Figure 6).

### 3.8. Theoretical Pharmacokinetic and Toxicological Profile of the Most Active Derivatives

We employed computational tools to assess the potential of the most active derivatives (**1a** and **1b**) as drug candidates and compared them with the marketed drug suramin (Table 2). Compound **1a** showed good intestinal absorption, unlike compound **1b** and suramin. To complement this analysis, we employed Lipinski’s “rule of five” and Veber rules. The latter one states that compounds with good oral bioavailability have tPSA ≤ 140 Å^2^ and RotB ≤ 10 [35] while the first one states that compounds must meet at least three of the following four criteria to exhibit high oral bioavailability: MW ≤ 500 Da, cLog ≤ 5, HBA ≤ 10, and HBD ≤ 5 [34]. Interestingly, both chalcones were approved according to these rules, but not suramin, indicating that they may show high oral bioavailability. Meanwhile, only compound **1a** was predicted to permeate the BBB. Also, we evaluated whether these compounds could inhibit CYP450 isoforms. Both compounds **1a** and **1b** were predicted to inhibit CYP2C19 and CYP2D6, but not CYP2C9 and CYP3A4.

Furthermore, we evaluated these compounds according to the GSK 4/400 rule. This rule determines that compounds with cLogP > 4 and MW > 400 Da are more likely to exhibit pharmacokinetic issues, based on several assays such as permeability, binding to plasma and tissue proteins, BBB permeability, and inhibitory profile towards a panel of CYP450 isoforms [36]. Compounds **1a**, **1b**, and suramin were approved according to this rule and present satisfactory pharmacokinetic properties.

Finally, we evaluated some toxicity parameters of these compounds, like mutagenicity, carcinogenicity, immunotoxicity, cardiotoxicity, hepatoxicity, and nephrotoxicity. Compound **1a** showed a potentially safe profile with no predicted toxicity risks, while compound **1b** raised some concerns regarding the toxic effects to immune cells and liver, like the marketed drug suramin. In addition, suramin was predicted to be nephrotoxic, which agrees with its experimental data. Consequently, our results reinforce the promising potential of these imine-chalcone derivatives as lead compounds for the treatment of MAYV infections.

## 4. Discussion

Chalcones are a class of compounds of natural origin that can also be obtained through organic synthesis. Our group has been exploring the antifouling potential of this chemical class, particularly imine-chalcones, such as compound **1a** [24]. In this study, we investigated the antiviral activity of compound **1a** and novel halogenated derivatives (**1b**–**1d**) against MAYV, because chalcones exhibit a wide range of biological activities, including antiviral activity, and halogens are present in a considerable number of FDA-approved drugs due to their ability to modify important pharmaceutical properties of drug candidates [37].

First, we demonstrated that the compounds did not present significant toxicity in Vero cells, with CC_50_ values ranging from 249.92 µM (**1c**) to >1000 µM (**1b** and **1d**). Chalcones are known to exhibit a wide cytotoxicity spectrum, with some subclasses displaying low toxicity while others, particularly natural compounds, are markedly cytotoxic depending on structural features and cell models [38,39,40]. Excitingly, our results showed that the imine-chalcones studied herein present a comparable cytotoxicity profile to the less cytotoxic subclasses previously reported. The high CC_50_ of compounds **1b** and **1d** (>1000 µM) is especially notable and indicates that the introduction of the imine functionality does not compromise cell viability in Vero cells. In fact, this value is substantially higher than that of natural chalcones such as Panduritin A, whose CC_50_ values are approximately 10 µM in multiple cell lines [39,40]. These findings reinforce the suitability of this scaffold for antiviral drug design.

Interestingly, all studied derivatives exhibited higher antiviral potency than the control drug suramin (EC_50_ = 38.97 µM; SI > 25.66), with the unsubstituted derivative **1a** being the most potent (EC_50_ = 12.15 µM), while the chlorine-substituted derivative **1b** showed the highest selectivity (SI > 59.10). Based on the obtained data, a preliminary structure-activity relationship (SAR) analysis can be performed. The unsubstituted derivative (**1a**) displayed the highest antiviral potency, reinforcing that the introduction of an imine group into the chalcone scaffold is favorable for the development of new compounds with anti-MAYV activity. Although the introduction of halogens at the *para* position of the chalcone B-ring reduced antiviral activity, a milder decrease was observed when a chlorine atom was introduced (**1b**). In contrast, this modification led to a significant reduction in cytotoxicity and an increase in selectivity. Moreover, we observed that the introduction of more electronegative halogens, such as fluorine (**1d**), resulted in decreased antiviral activity but improved selectivity, whereas the introduction of bulkier halogens, such as bromine (**1c**), reduced both antiviral activity and selectivity. These findings indicate that the stereoelectronic nature of *para*-substituents strongly modulates the antiviral profile of these derivatives. The synthesis and evaluation of novel derivatives bearing other substituents, such as hydrogen-bond donors and acceptors, electron-donating groups, and functional groups that modulate electron density through resonance, are essential to establish a more robust SAR in the future. Yet this study opens new avenues for the rational design of safer and more potent imine-chalcone derivatives against MAYV.

Indeed, chalcones have been widely investigated as antiviral agents across diverse viral families, and their potency varies according to structural modifications [20,39,41,42,43,44]. The micromolar activity range observed for the studied imine-chalcones (EC_50_ = 12.15–26.35 µM) is consistent with the antiviral profile reported for other synthetic chalcone derivatives. Notably, these EC_50_ values fall within the ranges previously reported for chalcones against diverse viral families, including retroviruses (23.4 pM–47.1 μM), respiratory RNA viruses such as Influenza and SARS-CoV-2 (0.8–50 μM), and other arboviruses such as DENV, ZIKV, and JEV (3.1–22 μM). Despite this, it is worth highlighting that direct comparison between these results may be limited, given the differences in the replication of viruses from different families and the different experimental conditions used in antiviral assays, including MOI, treatment time, and cell types. Still, this is the first report of antiviral activity of chalcones against MAYV. Thus, our findings expand the antiviral scope of chalcones to alphaviruses, reinforcing the versatility of this scaffold in targeting phylogenetically distinct viruses.

The most active compounds were selected for investigation of their mechanism of action through a set of different experiments. According to previous studies on alphavirus replication kinetics [25,26,27], and for comparison with compounds already described in the literature, we increased the MOI to 1 in these assays to shorten the replicative cycle time and specifically target each step of MAYV replication. Although this may influence the antiviral activity of the compounds, it is important to emphasize that both compounds **1a** and **1b** exhibited significant antiviral activity, as demonstrated across the different experiments. Initially, a time-of-drug-addition assay was performed to investigate at which stage of viral infection the compounds act. Our results indicated that both compounds **1a** and **1b** act primarily at early steps of MAYV replication but may also retain some activity when added at later stages. The temporal inhibition profile observed closely aligns with the profiles of some flavonoids that inhibit early events of replication of CHIKV, a related alphavirus. These flavonoids had maximal inhibition when added between 0 and 2 hpi, followed by a marked loss of activity after 4 hpi [45,46]. These findings reinforce that imine-chalcones may interfere with early events of the alphavirus replication cycle, which agrees with the fact that both groups belong to the same chemical class.

We further investigated the activity of these compounds at specific steps of the MAYV replicative cycle. Compounds **1a** and **1b** significantly inhibited viral adsorption (51.53% and 59.35% inhibition, respectively). Since chalcones and flavonoids share the same C6-C3-C6 polyphenolic scaffold [47], we compared the tested compounds with chalcones, flavonoid-rich extracts, and isolated flavonoids previously tested against MAYV. Lopes and colleagues [48] evaluated the effects of a hydroethanolic leaf extract from *Fridericia chica*, which is known to be rich in flavonoids, while a previous work of their group [49] assessed proanthocyanidin, a flavonoid isolated from the crude root fractions of *Maytenus imbricata*. Their results showed that these compounds did not inhibit MAYV adsorption, unlike the imine-chalcones. Yet, other chalcones can also inhibit the adsorption of different viruses. Zhang and colleagues [50] observed that the natural compound Isobavachalcone inhibits SARS-CoV-2 adsorption. Although SARS-CoV-2 and MAYV enter host cells through distinct mechanisms, this work reinforces the ability of chalcones to inhibit viral adsorption.

In addition to adsorption, we demonstrated that imine-chalcone derivatives impaired MAYV entry into host cells. Thottasseri and coworkers [51] recently demonstrated that a hybrid chalcone derivative containing a morpholinodiazenyl moiety blocks viral entry by disrupting clathrin-mediated vesicular trafficking, impairing capsid uncoating, interfering with endosomal acidification, and altering vesicle maturation. Considering that MAYV internalization occurs primarily via clathrin-mediated endocytosis, with possible involvement of caveolin-1-associated vesicles in some cellular contexts [52,53], the strong entry inhibition observed for compound **1a** suggests that these imine-chalcones likely interfere with one or more key events within this pathway that remains to be determined. Additional experiments with shorter treatment times may help elucidate the specific mechanism through which these compounds act.

Moreover, we observed that compound **1b**, but not **1a**, affected the release of new infectious MAYV particles. Conceição and colleagues [54] demonstrated that the flavonoid catechin EGCG inhibits MAYV replication at both early and late stages of the viral cycle, although the relative contribution of each step varies according to the host cell. Since chalcones and catechins share a common flavonoid-derived scaffold [55], members of this family possibly exhibit multifunctional antiviral properties. This is further supported by the inhibitory effect of EGCG on the early steps of CHIKV infection [56], highlighting the ability of compounds from the flavonoid family to interfere with distinct stages of alphavirus replication. We also investigated the direct effect of these derivatives on MAYV particles, but none of the compounds exhibited strong virucidal activity (approximately 10–20%), though a statistically significant reduction in viral titer was observed. The dilution of the viral suspensions strongly indicates that these effects are virucidal and not due to residual compounds in the cells during titration, since the final concentration was too low (0.1 µM) to produce significant antiviral or cytotoxic effects, as suggested in our initial experiments. Nevertheless, we hypothesized that the weak effects observed might contribute to their antiviral and time-course profiles, particularly at later times of the time-of-addition assay, because new infectious viral particles are released at 6–8 hpi [27]. Overall, our mechanistic studies suggest that chalcone derivatives primarily act at early stages of infection, such as virus-cell interaction, rather than directly on the viral particle. This is supported by our finding that compounds exert modest direct effects on viral particles, but cell pre-treatment resulted in significantly reduced viral replication.

Finally, we evaluated the potential of the most active derivatives (**1a** and **1b**) as drug candidates using computational tools. Both derivatives exhibited high oral bioavailability and are therefore suitable for oral delivery. Additionally, compound **1a** possibly crosses the BBB, enabling potential antiviral effects against neuroinfections and MAYV-induced neuropathology [57]. Since these compounds may interact with certain CYP isoforms, further experimental studies should be conducted to investigate possible drug–drug interactions, particularly in the context of polypharmacological therapies. Regarding toxicity risks, both compounds exhibited a theoretical safety profile comparable to suramin, an approved antiparasitic drug with in vitro anti-MAYV activity. Yet, in vitro and in vivo studies should be conducted to confirm their pharmacokinetic and toxicological properties. Overall, these predictions point to a promising profile for these derivatives as drug candidates and support the use of imine-chalcones as a new scaffold for the design and development of novel drugs for the treatment of MAYV infections.

## 5. Conclusions

In this study, four imine-chalcones were evaluated for their ability to inhibit MAYV replication. Overall, all compounds presented low cytotoxicity and a favorable antiviral profile, with EC_50_ values in the low micromolar range, highlighting **1a** and **1b** as the most promising. Our findings further indicated that these derivatives act predominantly during the early stages of the MAYV replication cycle, affecting both viral interaction with the cell surface and events immediately after entry. Compound **1b** could also interfere with processes related to the release of new viral particles, while both compounds showed low virucidal potential. In silico pharmacokinetic and toxicological studies further supported that both compounds **1a** and **1b** have a promising profile as drug candidates. Therefore, imine-chalcones expand the experimental antiviral repertoire against MAYV, as this is the first report describing their activity. Further in vitro and in vivo studies are essential to determine their suitability as antiviral drug candidates. Altogether, these data support imine-chalcones as promising lead candidates for further preclinical development against MAYV.

## Figures and Tables

**Table 1:** Cytotoxicity, antiviral, and selectivity of the synthesized imine-chalcone derivatives (**1a**–**1d**) and the reference compound suramin against MAYV replication in Vero cells. The values are presented as mean ± standard deviation.

Compounds	CC_50_ (µM) ^1^	Inhibition (%)	EC_50_ (µM) ^2^	SI ^3^
**1a**	382.37 ± 60.05	100 ± 0	12.15 ± 0.96	31.47
**1b**	>1000	88.91 ± 1.53	16.92 ± 1.86	>59.10
**1c**	249.92 ± 160.26	77.71 ± 1.20	26.35 ± 2.23	9.48
**1d**	>1000	86.12 ± 1.33	20.49 ± 2.03	>48.80
**Suramin**	>1000	95.96 ± 3.50	38.97 ± 6.38	>25.66

^1^ CC_50_ (50% cytotoxic concentration): Concentration needed to decrease 50% of cell viability. ^2^ EC_50_ (50% effective concentration): Concentration required to decrease 50% of virus yield. ^3^ SI (selectivity index): Calculated as the ratio between CC_50_ and EC_50_ values.

**Table 2:** Pharmacokinetic, toxicological, and drug-like properties of the most active derivatives (**1a** and **1b**) and the reference drug suramin predicted by computational tools. Prediction accuracy of the results obtained from admetSAR 2 webserver is shown in parentheses.

Compounds	1a	1b	Suramin
**HIA ^1^**	Yes	No	No
**BBB ^1^**	Yes	No	No
**iCYP2C9 ^2^**	No (0.88)	No (0.74)	No (0.88)
**iCYP2C19 ^2^**	Yes (0.66)	Yes (0.81)	No (0.89)
**iCYP2D6 ^2^**	Yes (0.63)	Yes (0.59)	No (0.93)
**iCYP3A4 ^2^**	No (0.90)	No (−0.90)	No (0.96)
**Lipinski “Ro5” ^1^**	Approved	Approved	Rejected
**Veber rule ^1^**	Approved	Approved	Rejected
**GSK 4/400 ^1^**	Approved	Approved	Approved
**Mutagenicity ^3^**	No (0.56)	No (0.54)	No (0.68)
**Carcinogenicity ^3^**	No (0.62)	No (0.55)	No (0.77)
**Immunotoxicity ^3^**	No (0.58)	Yes (0.70)	No (0.75)
**Cardiotoxicity ^4^**	No	No	No
**Hepatotoxicity ^4^**	No	Yes	Yes
**Nephrotoxicity ^2^**	No (0.81)	No (0.67)	Yes (0.58)

Parameters were calculated using different webservers, such as SwissADME ^1^, admetSAR 2 ^2^, ProTox-II ^3^, and pkCSM ^4^. HIA: Human intestinal absorption; BBB: Blood–brain barrier permeability; iCYP: inhibitor of CYP isoforms; Lipinski’s “Ro5”: Lipinski’s “rule of five”.

## Data Availability

The data underlying this study are available in the published article and its Appendix A.

## Abbreviations

The following abbreviations are used in this manuscript:

ATCC	American Type Culture Collection
BBB	Blood–brain barrier
CC_50_	Concentration needed to decrease 50% of cell viability
CHIKV	Chikungunya virus
cLogP	n-octanol-water partition coefficient
DENV	Dengue virus
DMEM	Dulbecco’s modified Eagle Medium
EC_50_	Concentration required to decrease 50% of the virus yield
FBS	Fetal bovine serum
GSK	GlaxoSmithKline
HBA	Number of hydrogen bond acceptor groups
HBD	Number of hydrogen bond donor groups
HIA	Human intestinal absorption
hpi	Hours post-infection
I/E ratio	Intracellular/extracellular ratio
MAYV	Mayaro virus
MOI	Multiplicity of infection
MW	Molecular weight
nsP	Non-structural proteins
PFU	Plaque-forming units
RotB	Number of rotatable bonds
SI	Selectivity index
SUR	Suramin
TLC	Thin-layer chromatography
tPSA	Topological polar surface area
VC	Virus control
ZIKV	Zika virus
